# Comparison of Antiretroviral Therapy Adherence Among HIV-Infected Older Adults with Younger Adults in Africa: Systematic Review and Meta-analysis

**DOI:** 10.1007/s10461-018-2196-0

**Published:** 2018-07-03

**Authors:** Najeebullah Soomro, Grace Fitzgerald, Janet Seeley, Enid Schatz, Jean B. Nachega, Joel Negin

**Affiliations:** 1 Sydney School of Public Health, The University of Sydney, Edward Ford Building (A27), Sydney, NSW 2006 Australia; 2 Rural Health Mildura, Monash University, Victoria, 3500 Australia; 3 Broken Hill University Department of Rural Health, The University of Sydney, Broken Hill, Australia; 4 London School of Hygiene & Tropical Medicine, London, UK; 5 MRC/UVRI Uganda Research Unit, Entebbe, Uganda; 6 School of Health Professions, University of Missouri, Columbia, USA; 7 Department of Medicine and Centre for Infectious Diseases, Stellenbosch University Faculty of Medicine and Health Sciences, Cape Town, South Africa; 8 Departments of Epidemiology, Infectious Diseases and Microbiology, University of Pittsburgh Graduate School of Public Health, Pittsburgh, PA USA; 9 Departments of Epidemiology and International Health, Johns Hopkins Bloomberg School of Public Health, Baltimore, MD USA

**Keywords:** HIV, Adherence, ART, Africa, Older adults, Systematic review

## Abstract

As access to antiretroviral treatment in low- and middle-income countries improves, the number of older adults (aged ≥ 50 years) living with HIV is increasing. This study compares the adherence to antiretroviral treatment among older adults to that of younger adults living in Africa. We searched PubMed, Medline, Cochrane CENTRAL, CINAHL, Google Scholar and EMBASE for keywords (HIV, ART, compliance, adherence, age, Africa) on publications from 1st Jan 2000 to 1st March 2016. Eligible studies were pooled for meta-analysis using a random-effects model, with the odds ratio as the primary outcome. Twenty studies were included, among them were five randomised trials and five cohort studies. Overall, we pooled data for 148,819 individuals in two groups (older and younger adults) and found no significant difference in adherence between them [odds ratio (OR) 1.01; 95% CI 0.94–1.09]. Subgroup analyses of studies using medication possession ratio and clinician counts to measure adherence revealed higher proportions of older adults were adherent to medication regimens compared with younger adults (OR 1.06; 95% CI 1.02–1.11). Antiretroviral treatment adherence levels among older and younger adults in Africa are comparable. Further research is required to identify specific barriers to adherence in the aging HIV affected population in Africa which will help in development of interventions to improve their clinical outcomes and quality of life.

## Introduction

Globally, there are 36.7 million people living with human immunodeficiency virus (HIV), of which 69% live in Africa [1]. UNAIDS estimated that in 2016 there were 5.8 (5.3–6.2) million people aged 50 years and older living with HIV, representing 17% of all adults aged 15 years and over living with HIV [2]. Their modelling data also predicts that the number of older adults living with HIV in low- and middle-income countries around the world will increase by 47% by 2020 and most of them will be living in sub-Saharan Africa (SSA).

Over the past decade there has been an increase in the access to life-prolonging antiretroviral therapy (ART), and factors such as improved HIV surveillance, clinician awareness of HIV, advancement of diagnostic techniques etc., have led to an increase in people aged 50 years and older living with HIV [3, 4]. As life expectancy in Africa is lower than developed countries around the world, WHO projects in Africa have categorized older adults as people aged 50 years and older [5]. This definition of older adults for reporting HIV incidence and ART adherence, in low and middle income countries, is well documented in the in published literature [6, 7].

With the increased life span, ageing population is becoming susceptible to other causes of ill health [8, 9], which in turn may affect their adherence to ART. Evidence shows that poor immunological response to ART is a surrogate of biological ageing [10]. The natural ageing process causes a decline in immunocompetence making older adults susceptible to both communicable and non-communicable diseases such as diabetes and hypertension when compared to young adults [11–13]. Therefore, understanding adherence of older adults to ART is paramount to develop strong HIV treatment programs.

Optimal adherence has both individual health benefits as well as wider public health benefits such as prevention of HIV endemic and improvement of mental health [14, 15]. Whereas poor adherence leads to incomplete viral suppression allowing disease progression which ultimately increases mortality [16, 17]. In 2014, UNAIDS called for the ambitious 90-90-90 targets with the aim of ending the global AIDS epidemic by improving surveillance, ART adherence and viral suppression in infected individual [18]. The program was aimed to achieve its goals by 2020 and one of the targets was to have 90% of all people receiving anti-retroviral treatment (ART) achieve viral suppression by 2020. This target can only be achieved if there is a strong emphasis on ART adherence. Despite the growing recognition that older people are increasingly infected with HIV [6, 19] and are accessing treatment, the literature on ART adherence among older adults is limited [20, 21]. This is particularly true in SSA where the burden of HIV among older adults is highest, and treatment and adherence challenges are greatest due to overburdened health services that are not set up to address older peoples’ needs.

A recent review has shown that the adherence to ART among older adults is higher than that of younger adults in developed countries [7]. There are reasons to believe this in the context developed countries, where older adults are likely to be financially stable and medically aware about their health. However, older adults in SSA might experience different barriers to adherence when compared to younger adults. These might include high rates of poverty, limiting money for transport and care [22, 23]; multi-morbidity with various chronic diseases leading to poly-pharmacy [24]; social isolation [25]; ageism or elder abuse [22]; and little understanding among health providers of older persons’ needs [22, 24]. Another important determinant of adherence in elderly is neurocognitive impairment. It has been shown that decline in executive functioning, motor functioning, and processing speed leads to suboptimal adherence in older adults [26]. Moreover, in high morbidity/mortality and high poverty areas, older adults often lose their caregivers and must provide care to others while suffering from age-related illness and frailty [27, 28]. As a result they may feel lonely, disconnected and despondent eventually becoming hopeless and demotivated to seek treatment [29, 30]. Depression is a surrogate of this sequel which has been shown to be a predictor for non-adherence (OR 2.54; 95% CI 1.65–3.91) [31].

A critical first step to improving adherence among older adults in Africa and thus helping to achieve the 90-90-90 targets is to understand current levels of treatment adherence. Currently there is no synthesis in the literature on the comparison of adherence rates for ART between older and younger adults living in Africa. Therefore, we conducted a systematic review and meta-analysis to examine adherence to ART in Africa among older adults compared to younger adults.

## Methods

### Definitions

For the purposes of this systematic review, we defined ART as any combination of antiretroviral therapy. Adherence pertains to the correspondence between a patient’s actual medication use and the regimen prescribed by a health care provider [32]. Adherence measurement may include the use of subjective measures i.e. self-reporting or objective measures such as pill-counts or medication possession ratio (MPR) [33]. Self-reporting by the use of any survey instrument was acceptable, including AIDS Clinical Trial Group (ACTG) adherence questionnaire [34]. The MPR was defined as the proportion of prescribed days’ supply obtained from the pharmacy during a specified observation period—generally the refill interval for a given prescription [35]. Older adults were defined as anyone aged 50 years and above. Individuals aged between 15 and 50 were categorized as young adults.

### Search Strategy and Selection Criteria

The study follows the PRISMA (Preferred Reporting Items for Systematic Reviews and Meta-Analyses) guidelines for search strategy, which aims to locate all published articles on the topic. The databases identified for this review were PubMed, Medline, Cochrane CENTRAL, CINAHL, Google scholar, and EMBASE, plus reference lists of relevant articles. The databases searched were based on the keywords: human immunodeficiency virus, antiretroviral therap*, compliance OR adherence, age* (elderly/older adults/adults), Africa. The search included all articles published from 1st Jan 2000 to 1st March 2016.

### Inclusion Criteria

The review considered studies that included people on ART, included data on participants aged 50 years and above and compared them to a younger cohort, that were conducted in Africa and that included data on adherence. We included cross-sectional studies, prospective studies, retrospective studies, case control studies and randomized control trials. We considered studies that covered data on adherence to ART using a wide variety of measures. We excluded case reports, reviews and publications in languages other than English.

### Data Extraction and Analysis

**Fig. 1:**
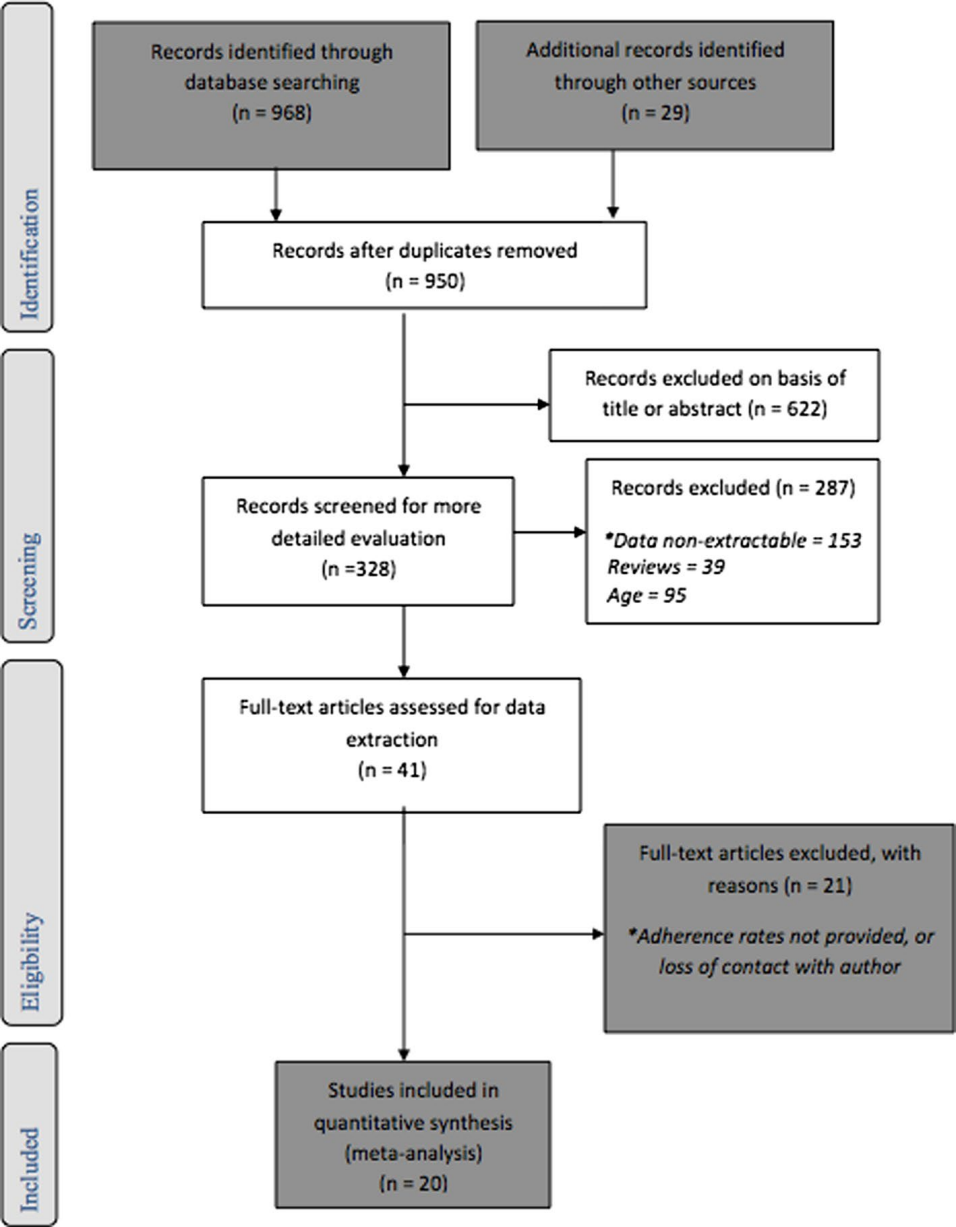
Prisma flow chart of study selection process. *Where articles only included children or did not include older adults

The study-screening process adhered to PRISMA recommendations. After duplicates were removed from the independent searches, articles were excluded per the identified criteria for study selection. It is illustrated in Fig. 1.

Where a screened study made reference to adherence but did not present data on adherence rates in that cohort, author GF contacted the corresponding author to ask if appropriate data were available. Similarly, if a study presented adherence rates that were not stratified into age groups, author GF contacted the author to assess the possibility of stratifying the relevant data.

### Data Extraction

Quantitative data was extracted by NS and GF using the standardized data extraction tool from JBI-MAStARI [36] (Appendix 1). Information was extracted pertaining to study title, author names, and publication year, research design, and adherence measures. Sample data relating to number of participants, follow up periods and number of participants (total and in both the older and younger age groups) was collected. The outcome measures extracted were the proportion of adherence in the older and younger age groups, recorded as number of adherent participants in each group, and proportion of that group who were adherent.

### Assessment of Methodological Quality

Given the use of both randomized and non-randomized studies in our analysis, methodological quality was assessed using a modified version of the Downs and Black Framework [37]. Studies were independently scored using 24 quality criteria. As most studies included in the analysis were either cohort studies or cross-sectional studies, questions pertaining to Randomization or RCTs were excluded (Appendix 1 describes the items and their assessment). With the exception of one item scored from 0 to 2 points, all items were scored as follows: 1 = yes (1 point), – = no (0 points), ? = don’t know (0 points).

### Meta-analysis

We pooled studies using Comprehensive Meta-Analysis software v 3.3.070 (2014). All results were subjected to double data entry check. The odds ratio (OR) for each study was calculated and we used a random-effects (DerSimonian-Laird) approach for pooling. We examined heterogeneity using the I^2^ value. We first pooled all randomized trials and then added observational studies. We examined differences in treatment effects according to study designs.

## Results

### Study Characteristics

From 968 studies initially identified, 328 were screened for detailed evaluation. Between August and November 2016, 169 authors were contacted, and 22 of those responded to provide further information. Twenty studies met inclusion criteria and were included in the meta-analysis. Figure 1 presents the search results and the number of studies extracted from research databases. The 20 studies identified for inclusion included eight cohort studies, two retrospective cohort studies, five cross-sectional studies, four randomized trials, and one case–control study. In terms of geographical area six studies used data from Zambia, three from Cote D’Ivoire, two each from Uganda, Tanzania, Kenya, and South Africa. Table 1 depicts the characteristics of the included studies.

**Table 1:** Summary of included studies

Author	Country	Type of study	Length of follow up	Sample size	n ≥50 years	Adherence n (%) <50 years of age	Adherence n (%) ≥50 years of age
Diabate et al. [67]	Cote D’Ivoire	Retrospective cohort	3–6 months	614	52 (8.5%)	435 (77.4%)	41(78.85%)
Carlucci et al. [68]	Zambia	Cohort	N/A	409	71 (17.4%)	295 (87.3%)	60 (84.5%)
Fielding et al. [69]	South Africa	Cohort	12 months	1439	225 (15.6%)	1065 (87.77%)	195 (86.7%)
Birbeck et al. [70]	Zambia	Retrospective study	12 months	255	29 (12.9%)	130(57.3%)	17 (59.7%)
Pirkle et al. [71]	Burkina Faso	Single-arm pilot (cohort)	1 month	606	45 (7.5%)	362 (65%)	29 (64%)
Stubbs et al. [72]	Mozambique	Pharmacy record review	6 months	426	46 (10.8%)	273 (71.8%)	32 (69.6%)
Jaquet et al. [73]	Benin, Côte d’Ivoire and Mali	Cross-sectional	N/A	2065	277 (11%)	1628 (91.1%)	246 (88.8%)
Lester et al. [74]	Kenya	Randomized clinical trial	12 months	538	43 (8%)	282 (57%)	19 (44.2%)
Watt et al. [75]	Tanzania	Cross sectional	N/A	339	34 (10%)	291(95.4%)	29 (85.3%)
Chung et al. [76]	Kenya	Prospective cohort	18 months	347	28 (8%)	221 (69.3%)	16 (57.1%)
Kunutsor et al. [77]	Uganda, Zambia	Randomised controlled trial	13 months	174	20 (11.5%)	139 (90.3%)	18 (90%)
Messou et al. [78]	Cote D’Ivoire	Prospective cohort	12 months	996	95 (9.5%)	384 (34%)	39 (41%)
Ncaca et al. [79]	South Africa	Case-control	16 months	244	10 (4.5%)	190 (81.2%)	10 (100%)
Mbuagbaw et al. [80]	Cameroon	Randomized trial	6 months	160	33 (20.6%)	88 (69.3%)	23 (69.7%)
Annison et al. [81]	Ghana	Cohort	18 months	280	37 (13.2%)	205 (84.4%)	34 (92%)
Kiwuwa-Muyingo et al. [82]	Uganda, Zambia	Randomised controlled trial	12 months	2960	191 (6.5%)	800 (28.9%)	50 (26.2%)
Negash et al. [83]	Ethiopia	Cross sectional	N/A	355	35 (10%)	236 (73.7%)	25 (71.4%)
Jones et al. [84]	Zambia	Cohort	12 months	249	25 (10%)	182 (81.3%)	17 (68%)
Vinikoor et al. [39]	Zambia	Cross Sectional study	24 months	92,130	6281 (6.8%)	51,902 (60.5%)	3869 (61.6%)
Muya et al. [38]	Tanzania	Prospective cohort	60 months	44204	4863 (11%)	39342 (85.2%)	4211 (86.6%)
Pooled data				148,819	12,401 (8.3%)	92,705 (68%)	8947 (72.15%)

Table 2 describes different adherence measures used in the included studies. Adherence was most commonly measured by patient self-reported questionnaires, or by pill counts systems. Two studies calculated MPR, and another two constructed an adherence measure based on clinical contact. The most frequent definition of adherence was equal or greater than 95% compliance with the prescribed regime of ART.

**Table 2:** Summary of adherence measures

Author	Adherence measure	Adherence cut off
Diabate et al. [67]	Self report with ACTG^a^	≥ 95%
Carlucci et al. [68]	Pill count	≥ 95%
Fielding et al. [69]	Self report	100% reported adherence in last 3 days
Birbeck et al. [70]	Attendance at clinic and self report	Attended all scheduled ART clinic visits with no lapse in drug collection and documentation
Pirkle et al. [71]	Self-report	N/A
Stubbs et al. [72]	Pill count	≥ 90%
Jaquet et al. [73]	Self report with ACTG^a^	≥ 95%
Lester et al. [74]	Self report—asked how many pills missed in 30 days	≥ 95%
Watt et al. [75]	Self report with ACTG^a^	≥ 95%
Chung et al. [76]	Pill count	≥ 95%
Kunutsor et al. [77]	Pill count	≥ 95%
Messou et al. [78]	Pill count	≥ 95%
Ncaca et al. [79]	Medication possession ratio	≥ 95%
Mbuagbaw et al. [80]	Self report using visual analogue scale	≥ 95%
Annison et al. [81]	Self report	Patients without treatment interruptions
Kiwuwa-Muyingo et al. [82]	Pill count and structured questionnaire	Good adherence defined as “did not miss a dose in last month”
Negash et al. [83]	Self report of medication doses taken divided by medication doses prescribed	≥ 95%
Jones et al. [84]	Self report using ACTG^a^	Adherence over last two weeks
Vinikoor et al. [39]	Medication Possession ratio	≥ 95%
Muya et al. [38]	Compliance with pick-up visits	≥ 95%

^a^AIDS Clinical Trial Group Adherence Questionnaire [34]

### Quality Assessment

Individual study scores are summarized in Appendix 2. Studies were considered ‘‘high quality’’ if they scored 60% of the maximum score.

### Meta-analysis

**Fig. 2:**
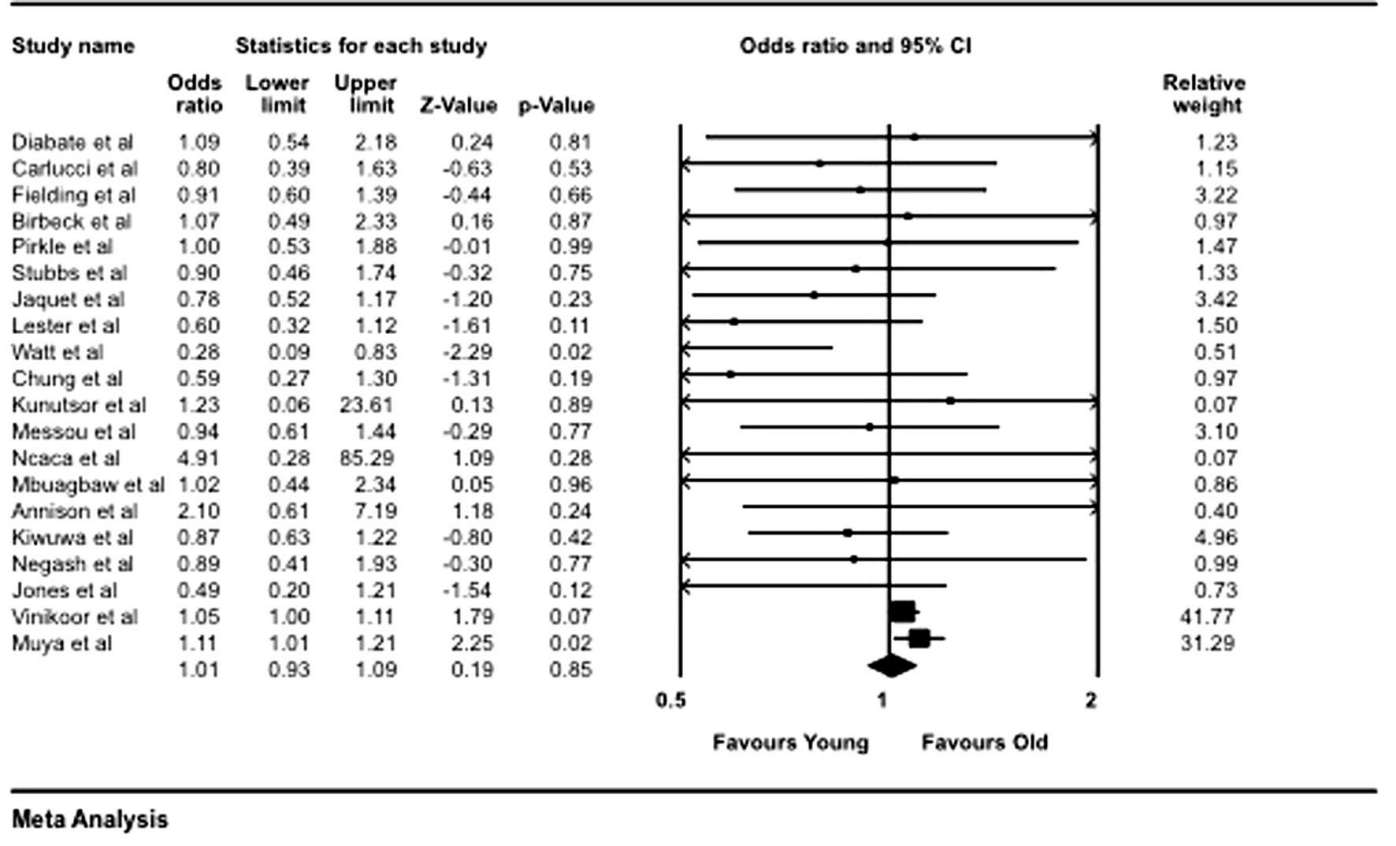
Forest plot indicating pooled effect of adherence among older adults compared with younger adults

The overall cohort of subjects for the 20 included studies was 148,819. The pooled data showed that of the 12,401 older adults, 8947 (72.15%) were considered adherent; and of the 136,351 young adults, 92,705 (68.03%) were adherent. The meta-analysis yielded a pooled OR of 1.01 (95% CI 0.93–1.09), showing no difference in the likelihood of adherence between older and younger adults p = 0.85 (Fig. 2).

**Fig. 3:**
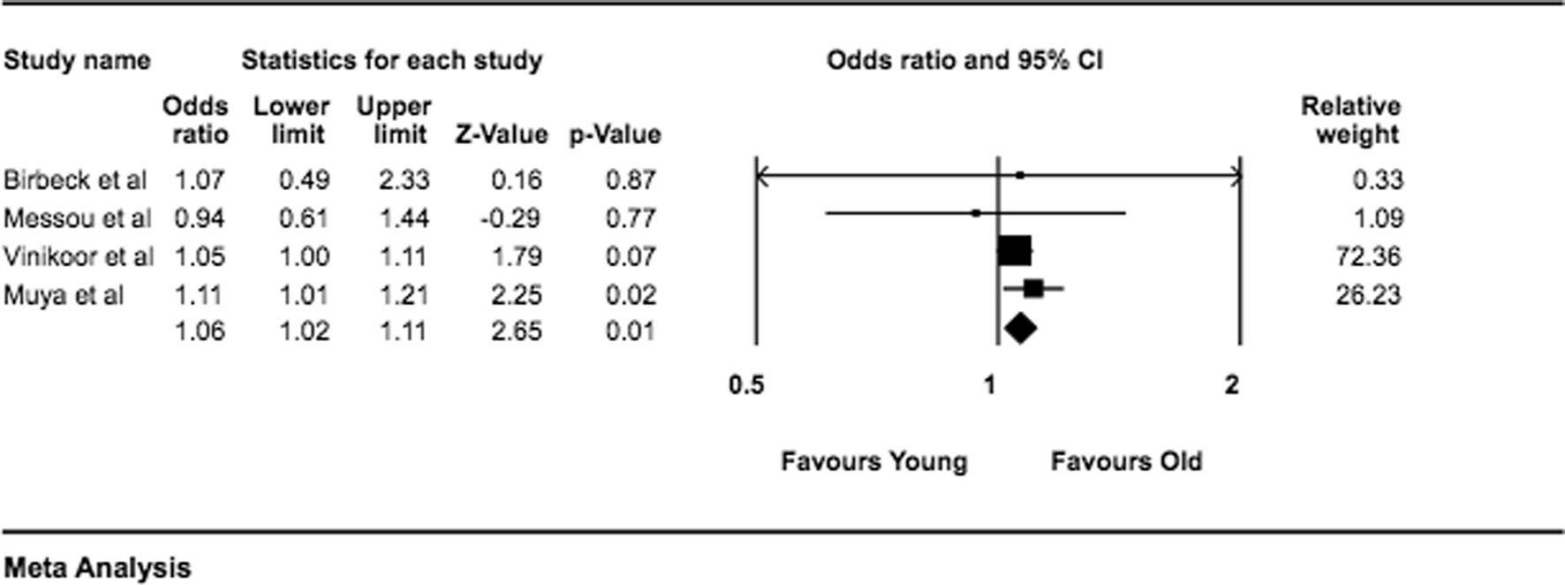
Forest plot indicating sub-group analysis of studies using MPR or clinician contact as adherence measure

Due to the heterogeneity of methods used for measuring adherence, a sub-group analysis was conducted by grouping subsets of studies that used similar methodology for measuring adherence. Four studies used MPR and clinician counts, six used pill counts and seven used self-reports for measuring adherence. When pooled by objective measures of adherence monitoring i.e. MPR and clinician counts, the analysis showed significantly higher proportions of adherence among older adults compared with their younger peers OR 1.06 (95% CI 1.02–1.11). However, when data from the six studies using objective measure of pill counts was pooled, there was no difference between the two groups OR 0.84 (95% CI 0.65–1.09). Meanwhile pooling of studies using subjective measure of adherence ‘self-report’ showed no significant difference between the older and younger cohorts OR 0.92 (95% CI 0.72–1.18). Additional sub-group analyses can be found in Appendix 3 (Fig. 3).

A further analysis on the effect of study design was carried out. The studies were sub-divided into four groups, RCTs (4), cross-sectional studies (5), prospective cohort studies (7) and others (retrospective cohort, case–control, etc.) (3). When the data from the four RCTs was analysed as a group, the results showed no statistically significant difference between the two groups OR 0.83 (95% CI 0.63–1.09). The other results for sub-group analysis based on study type were non-significant, cross-sectional studies OR 0.89 (95% CI 0.69–1.15), prospective cohort studies [OR 0.87 (95% CI 0.69–1.01)] the analysis of the other study types OR 1.13 (95% CI 0.68–1.89) see Appendix 3. These results showed that study design was not an important moderator to affect adherence patterns.

The included studies in the analysis had different weights, this weighing is a factor of their sample sizes and variance [36.] However, two studies [38, 39] had a very high relative weight, forming almost 90% of the pooled results. When these studies were excluded the remaining 18 studies showed younger adults were more likely to be adherent OR 0.85 (95% CI 0.73–0.98), p = 0.03 (Appendix 3).

## Discussion

Our systematic review shows that ART adherence among older people is similar to that of younger people in SSA. Despite a general neglect of programs for older adults living with HIV in Africa and despite issues of poverty, social exclusion, lack of support and mobility challenges, adherence is not statistically different to that of younger people. A 2013 meta-analysis by Ghidei and colleagues identified 12 studies from non-African settings that contained data on older adult adherence compared to younger counterparts [7]. They found that older age reduced the risk of non-adherence by 27% in the studies from the US, Italy, United Kingdom, Canada and Brazil. Our study found no such difference in their adherence. However, this review included studies that used a variety of different measures of adherence each of which have their own strengths and weaknesses [40]. For example, self-report is a practical tool in low-resource settings, but can overestimate adherence [41]. Pharmacy refill adherence measures such as pill counts and calculation of the medication possession ratio can be impractical and do not measure whether patients are actually taking their medication, although are less prone to reporting bias than self-report [42, 43]. Clinical contact measures require a clinician to classify adherence as good or poor, and thus subject to assessment bias and difficult to standardize [43]. All three included measures have been demonstrated to be significantly predictive of detectable viremia [44].

We found that when MPR or clinician counts were used as the measure of adherence, older adults were more adherent than of younger adults. The reasons for this is unclear; however, it could be argued that pharmacy refill-based adherence monitoring tools (e.g. MPR) are better adherence measures than self-report [45]. It is well accepted that there is no gold standard to measure ART adherence [46]. Yet a number of available adherence methods such as self-reports, pill counts, pharmacy records, self-report electronic monitoring, and therapeutic drug levels can be used depending on the setting or the available resources. Self-reports and pharmacy refill are the most common approaches used in Africa due to the low-cost and simplicity. Common limitations include poor sensitivity to detect non-adherence (e.g. social desirability bias for self-report or pill dumping for pill count) but good specificity for pharmacy refills [42]. Therefore, pharmacy refills may accurately capture older persons’ adherence behaviour, making it a better measure for this population.

Another interesting finding in our analysis was when two studies with the largest weights were discounted from the pooled analysis, younger adults were significantly more adherent than older adults p = 0.03. The key differentiating characteristic of these two studies was that their ‘length of follow up’ was greater than the other studies i.e. 24 and 60 months respectively. This may mean that longitudinally older adults may be more likely to be adherent to an ART program. Adherence in older adults is complex and the next challenge is to better understand the individual, interpersonal, community and structural factors behind adherence, and most importantly, the levers by which adherence could be improved. For example, Shubber and colleagues’ in a recent systematic review on patient-reported barriers identified a number of obstacles including depression, stigma and health-service related barriers such as distance from facility [47]. Cultural norms and personal beliefs have been acknowledged as key predictors for medication adherence in elderly [48]. In the indigenous culture illness is sometimes considered as fate, or a condition whose control is held by an external locus [49, 50]. These believes combined with the likelihood that older adults living in Africa are less likely to be exposed to contemporary western medicine and more likely to have deeper roots in their tradition, when compared to their younger counterparts, makes adherence a challenge. A solution to that can be the inclusion of patient centred pharmaceutical counselling, this may be an important tool to improve their adherence. However, the evidence for effectiveness of pharmaceutical counselling is from developed countries [51, 52], and may not be easily translatable in the context of low-resource countries in Africa. In these settings, the interaction between physical and mental health becomes particularly important [30], and future studies should have a special focus on this aspect.

In developed countries older adults show less attrition on ART [53], and the CD4 reconstitution is low [54–57], thus increasing the importance of early access and strong adherence to reducing mortality. Past studies have explored the impact of depression on adherence in Africa [58] as well as exploring the impact of community-based adherence interventions [59–61]. A number of peer-led interventions, such as medical companions or support groups for people living with HIV, have proven successful in improving ART access and adherence in SSA [62, 63] but nearly all exclusively target people under 50 years of age [64]. Therefore, prospective programs need to have a focus on older adults living in low-resource or developing countries.

Understanding reasons for late presentation and mechanisms for addressing that shortcoming would be critical to improving outcomes among older people. Overall, however, there have been very limited HIV-related behavioral interventions among older people [65] which has limited the development of appropriately and specifically targeted programs. In order to achieve global HIV goals, the health providers must strive to improve the quality of care and quality of outcomes among all people living with HIV. While older adults have long been neglected, there are promising signs that their specific needs are now being recognised and that appropriate programs will emerge to ensure that older people remain productive members of their communities.

### Limitations

The diversity of methods used to measure adherence is an inherent challenge in systematic review and meta-analysis as we have undertaken. Further, the lack of standardised conversion of continuous measurements of adherence rates into dichotomous or ordinal data weakens any direct comparison between different adherence levels across studies. Some confounding factors such as chronological age, gender, urban–rural status, overall health, financial status, and mobility could not be accounted for while doing the analysis.

The core challenge in this review was that individual studies varied in their definition and measurement of adherence. To deal with this limitation the authors included an additional variable based sub-analysis, stratified by adherence definition. This sub-grouping and pooling of similar studies may eliminate some confounders for on the variability of methodologies among the studies. Similarly, the authors acknowledge that dichotomising age may have had implications on adherence rates. However, the authors ensured that the age cut-off used are consistent with the WHO definition of older and younger adults in Africa [5]. Another limitation may have been the length of follow-up among the studies, as shorter studies may have a higher adherence rate than longer studies. To understand if there was an effect we ran a linear regression analysis between the length of the studies and adherence rates, and noted that there was no significant difference among the studies due to this variable (β = 0.008, p > 0.05) (Appendix 4). Despite literature suggesting that adherence changes over time [66], the fact that our analysis data didn’t show significance can be attributed to the fact that both older and younger adults maintain similar attrition rates and age is not a contributory factor. This further supports our results that there is no significant difference in among the two age categories.

In the future, studies need to be designed with a standardized measure of adherence in order to improve validity and generalizability. The authors further recommend that studies track adherence data at set time points, for example every three months, this will allow standardized comparison while pooling data for future analysis.

## Conclusions

This systematic review and meta- analysis found ART adherence levels were similar among older and younger adults in Africa. Further research might identify specific barriers to adherence in the HIV affected populations, and targeted interventions to improve clinical outcomes and quality of life when those affected by HIV grow older.

## Appendix 1: Quality Assessment Items

**Table:**

Quality assessment item	Maximum points awardable
Reporting (9 items)	
Is hypothesis/aims clearly described?	1
Are the main outcomes to be measured clearly described?	1
Are the characteristics of the patients included in the study clearly described	1
Are the interventions of interest clearly described?	1
Are the distributions of principal confounders in each group of subjects to be compared clearly described?	2
Are the main findings of the study clearly described?	1
Does the study provide estimates of the random variability in the data for the main outcomes?	1
Have all important adverse events that may be a consequence of the intervention been reported?	1
Have the characteristics of patients lost to follow-up been described?	1
Have actual probability values been reported (e.g. 0.035 rather than <0.05) for the main outcomes except where the probability value is less than 0.001?	1
External validity (3 items)	
Were the subjects asked to participate in the study representative of the entire population from which they were recruited?	1
Were those subjects who were prepared to participate representative of the entire population from which they were recruited?	1
Were the staff, places, and facilities where the patients were treated, representative of the treatment the majority of patients receive?	1
Bias (7 items)	
If any of the results of the study were based on “data dredging”, was this made clear?	1
In trials and cohort studies, do the analyses adjust for different lengths of follow-up of patients, or in case-control studies, is the time period between the intervention and outcome the same for cases and controls?	1
Were the statistical tests used to assess the main outcomes appropriate?	1
Was compliance with the intervention/s reliable?	1
Were the main outcome measures used accurate (valid and reliable)?	1
Confounding (6 items)	
Were the patients in different intervention groups (trials and cohort studies) or were the cases and controls (case-control studies) recruited from the same population?	1
Were study subjects in different intervention groups (trials and cohort studies) or were the cases and controls (case-control studies) recruited over the same period of time?	1
Were losses of patients to follow-up taken into account?	1
Power (1 item)	
Does the study describe its calculation of power in order to demonstrate a clinically significant effect?	1
Total	23

## Appendix 2: Quality Assessment Scores

**Table:**

Reporting item	Reporting (max 11 points)	External validity (max 3 points)	Bias (max 5 points)	Confounding (max 3 points)	Power (max 1 point)	Total (max 23 points)	Total %
Author							
Diabate et al. [67]	8	2	4	1	0	15	65
Carlucci et al. [68]	8	2	5	2	0	15	65
Fielding et al. [69]	10	1	5	3	0	17	74
Birbeck et al. [70]	8	1	5	3	0	15	65
Pirkle et al. [71]	7	2	5	2	0	14	61
Stubbs et al. [72]	11	2	5	2	0	18	78
Jaquet et al. [73]	8	3	5	0	0	16	70
Lester et al. [74]	10	3	5	3	1	20	87
Watt et al. [75]	9	1	5	2	0	15	65
Chung et al. [76]	10	3	5	3	1	20	87
Kunutsor et al. [77]	9	3	4	3	0	17	74
Messou et al. [78]	8	2	5	2	0	15	65
Ncaca et al. [79]	9	3	5	2	0	17	74
Mbuagbaw et al. [80]	9	2	5	3	1	18	78
Annison et al. [81]	7	2	5	2	0	14	61
Kiwuwa-Muyingo et al. [82]	10	2	5	3	0	18	78
Negash et al. [83]	8	2	5	2	1	16	70
Jones et al. [84]	8	2	5	2	0	15	65
Vinikoor et al. [39]	8	2	5	3	0	16	70
Muya et al. [38]	9	2	5	2	0	16	70

## Appendix 3: Additional Forest Plots—Sub-Group Analyses

Analysis of studies using self-report as adherence measure.

Analysis of studies using pill count as adherence measure.

Analysis excluding two highest weighted studies.
